# Identification and Characterization of a Novel Galactofuranose-Specific β-D-Galactofuranosidase from *Streptomyces *Species

**DOI:** 10.1371/journal.pone.0137230

**Published:** 2015-09-04

**Authors:** Emiko Matsunaga, Yujiro Higuchi, Kazuki Mori, Nao Yairo, Takuji Oka, Saki Shinozuka, Kosuke Tashiro, Minoru Izumi, Satoru Kuhara, Kaoru Takegawa

**Affiliations:** 1 Department of Bioscience and Biotechnology, Faculty of Agriculture, Kyushu University, 6–10–1 Hakozaki, Fukuoka, Japan; 2 Department of Applied Microbial Technology, Faculty of Biotechnology and Life Science, Sojo University, Kumamoto, Japan; 3 Graduate School of Environmental and Life Science, Okayama University, Okayama, Japan; Hans-Knoell-Institute (HKI), GERMANY

## Abstract

β-D-galactofuranose (Gal*f*) is a component of polysaccharides and glycoconjugates and its transferase has been well analyzed. However, no β-D-galactofuranosidase (Gal*f*-ase) gene has been identified in any organism. To search for a Gal*f*-ase gene we screened soil samples and discovered a strain, identified as a *Streptomyces* species by the 16S ribosomal RNA gene analysis, that exhibits Gal*f*-ase activity for *4*-nitrophenyl β-D-galactofuranoside (*p*NP-β-D-Gal*f*) in culture supernatants. By draft genome sequencing of the strain, named JHA19, we found four candidate genes encoding Gal*f*-ases. Using recombinant proteins expressed in *Escherichia coli*, we found that three out of four candidates displayed the activity of not only Gal*f*-ase but also α-L-arabinofuranosidase (Ara*f*-ase), whereas the other one showed only the Gal*f*-ase activity. This novel Gal*f*-specific hydrolase is encoded by ORF1110 and has an optimum pH of 5.5 and a Km of 4.4 mM for the substrate *p*NP-β-D-Gal*f*. In addition, this enzyme was able to release galactose residue from galactomannan prepared from the filamentous fungus *Aspergillus fumigatus*, suggesting that natural polysaccharides could be also substrates. By the BLAST search using the amino acid sequence of ORF1110 Gal*f*-ase, we found that there are homolog genes in both prokaryotes and eukaryotes, indicating that Gal*f*-specific Gal*f*-ases widely exist in microorganisms.

## Introduction

β-D-galactofuranose (Gal*f*) is a constituent of polysaccharides and glycoconjugates that are present on the surface of the cell wall in many pathogenic bacteria and eukaryotes [1,2]. Gal*f* is present in bacteria, filamentous fungi, trypanosomatids and nematodes, but not in yeasts nor in mammals [3,4]. Because Gal*f* is known to be immunogenic to mammals [5–8], it is now a target molecule for anti-fungal reagents to suppress pathogenicity [1,2,4,9,10].

In certain filamentous fungi, Gal*f* is found in galactomannan (GM), galactomannoproteins modified with *N*-glycans and *O*-glycans and glycolipids [1,2,11–16]. Filamentous fungi enzymes involved in Gal*f*-containing oligosaccharide synthesis have been well studied, especially in *Aspergillus*. For instance, in the model filamentous fungus *Aspergillus nidulans*, at the initial step of Gal*f*-sugar chain synthesis, UDP-glucose, which is a donor substrate for α- and β-glucan synthesis, is converted to UDP-galactopyranose (UDP-Gal*p*) by the UDP-glucose-4-epimerase UgeA [17]. Then, UDP-Gal*p* is converted to UDP-Gal*f* by the UDP-Gal*f* mutase UgmA (GlfA in *Aspergillus fumigatus*) [18–20]. These reactions occur in the cytoplasm. UDP-Gal*f* is subsequently transported into the Golgi lumen by the UDP-Gal*f* transporter UgtA (GlfB in *A*. *fumigatus*) which localized in the Golgi membrane [21,22]. To identify a Gal*f* transferase gene in *A*. *nidulans*, we previously conducted reverse-genetics and biochemical approaches. We identified a gene named *gfsA* that encodes the Gal*f* transferase localized to Golgi which function is to attach UDP-Gal*f* onto the *O*-glycan chain [23]. Δ*ugmA*, Δ*ugtA* and Δ*gfsA* strains exhibit retarded hyphal morphology, suggesting that the Gal*f* biosynthetic pathway is crucial for cell growth [19,22,23].

While the molecular mechanisms of the biosynthesis of Gal*f*-containing sugar chains have been analyzed, enzymes involved in degradation and metabolism of Gal*f*-oligosaccharides are not well known [1]. One such enzymes is β-D-galactofuranosidase (Gal*f*-ase), which can release Gal*f* from polysaccharides and glycoconjugates. There are reports about the purification of exo- and endo-Gal*f*-ases from the culture supernatant of several microorganisms [24–29]. However, no Gal*f*-ase gene has been identified. α-L-arabinofuranosidase (Ara*f*-ase), which hydrolyzes α-L-arabinofuranoside (Ara*f*), is structurally similar to Gal*f*, and Ara*f*-ase-encoding genes have been identified in *Aspergillus* species [10,30–37]. In *Aspergillus niger*, Ara*f*-ases, which belong to glycosyl hydrolase family 51 (GH51) and 54 (GH54), have both Ara*f*-ase and Gal*f*-ase activities [30]. However, no gene encoding a Gal*f*-ase-specific enzyme has been reported yet [38].

In this study, we screened soil samples for microorganisms that exhibit Gal*f*-ase activity. The screen allowed us to identify a novel gene that encodes a Gal*f*-ase-specific enzyme, which does not exhibit any Ara*f*-ase activity.

## Materials and Methods

### Microorganism, cultivation and microscopy

Bacteria were isolated from soil in Kagawa University, Japan. Since the area of the university is public, no specific permission was required to collect samples that did not include any endangered nor protected species. The isolated strain JHA19 (material number, QM2015–0042) has been deposited in the Material Management Center (MMC; http://mmc-u.jp/en/). YMG medium (0.4% yeast extract, 1% malt extract, 0.4% glucose and 2% agar, pH 7.3) was used for bacterial growth on plates and in liquid cultures, which were performed at 30°C with shaking at 200 rpm. Cells of the isolated strain JHA19 were cultured in YMG liquid medium for 3 days and observed under an Eclipse 80i microscope (Nikon) with a Plan Apo 100x/1.40 NA oil objective lens (Nikon). Images were acquired with a CoolSNAP EZ CCD camera (Photometrics) and the software MetaVue (Molecular Devices).

### Preparation of *4*-nitrophenyl β-D-galactofuranoside

*4*-nitrophenyl β-D-galactofuranoside (*p*NP-β-D-Gal*f*) was synthesized as described previously [39,40] with some modifications as follows: Galactose (1.80 g, 10 mmol) was stirred at 70°C in pyridine (30 mL, 3.7 mmol) for 1 h, and then acetylated with acetic anhydride (5.82 mL, 61.5 mmol) for 12 h. The mixture was purified conventionally, to give per-*O*-acetyl-α,β-D-Gal*f* (syrup, 3.78 g, 97% yield, including per-acetyl Gal*p* as isomer). *p*NP (3.92 g, 28.2 mmol) was added to a solution of per-*O*-acetyl-α,β-D-Gal*f* (3.78 g, 9.7 mmol, including per-acetyl Gal*p*) in dry CH_3_CN (50 ml) cooled to 0°C. After 10 min of stirring, BF_3_-Et_2_O (3.8 mL, 30 mmol) was added. After 24 h, the reaction mixture was diluted with ethyl acetate, extracted with saturated NaHCO_3_ aq. until neutralization was completed, washed brine, and dried with MgSO_4_. After filtration, the solvent was evaporated under vacuum, and then syrupy liquid *p*NP-2,3,5,6-tetra-*O*-acetyl-β-D-Gal*f* was obtained. For analytical purposes, a part of the sample was purified by column chromatography, however most of sample was deacetylated without purification. Syrupy material *p*NP-2,3,5,6-tetra-*O*-acetyl-β-D-Gal*f* was suspended in 1.0 mol/L CH_3_ONa in CH_3_OH (30 ml), and stirred at room temperature during 12 h. The reaction mixture was evaporated under vacuum, and purified by silica-gel column chromatography (CHCl_3_/CH_3_OH, 4:1) to give colorless solid (*p*NP-β-D-Gal*f*, 0.24 g, 8% yield, over two steps from per-*O*-acetyl-α,β-D-Gal*f* to *p*NP-β-D-Gal*f*).

### Enzyme assay

Gal*f*-ase and Ara*f*-ase activity was determined using *p*NP-β-D-Gal*f* or *p*NP-α-L-Ara*f* as a substrate, respectively. The enzyme solution was prepared in 45 μL, which was mixed with 2.5 μL of 10 mM substrate and 2.5 μL of 1 M acetate buffer, pH 4.5. After incubation for the appropriate time at 37°C, 50 μL of 1 M sodium carbonate was added to terminate the reaction, and the liberated *p*NP was determined from absorbance at 405 nm. One unit (U) of enzyme activity was defined as the amount of enzyme required to liberate 1 mmol of *p*NP per min [24,28]. The activity of exoglycosidases was assessed using appropriate *p*NP-glycosides (α-D-Xyl and β-D-Xyl from Seikagaku; the others from Sigma).

### Preparation of genomic DNA

Genomic DNA of strain JHA19 was extracted as described previously with certain modifications [41]. After culture in 100 mL YMG medium at 30°C for 1 week, the culture of the strain JHA19 was centrifuged at 5000 rpm for 15 min and the cell pellet was resuspended in 5 mL TE_10_ (10 mM Tris-HCl, 10 mM EDTA, pH 8.0). After addition of 10 mg lysozyme (Wako) and 10 mg achromopeptidase (Wako), the cell suspension was incubated at 37°C for 20 min. The resultant sample was added with 100 μL TE_10_, 2.5 ml EDTA (0.5 M, pH 8.0), 1.25 mL 10% (w/v) SDS and 125 μL proteinase K (20 mg/mL) (Wako) and incubated overnight at 37°C. After another incubation at 65°C for 5 min, 20 mL TE_10_ was added. Ten mL of the resultant sample was taken and mixed with 20 mL TE_10_, 2 mL 3 M sodium acetate and 20 mL phenol/chloroform (1:1, v/v) by gently rotating for 30 min. After centrifugation at 4500 rpm for 20 min, the aqueous phase was divided into two tubes. Each tube was added with 2.5 volume 100% ethanol and centrifuged at 4500 rpm for 10 min. The pellet was dried and suspended in 10 mL TE. Those two genomic DNA suspension tubes were combined into one tube, which was added with 10 μL RNase (10 mg/mL) and incubated at 37°C for 30 min. The resultant sample was added with 200 μL 10% (w/v) SDS and 50 μL proteinase K (10 mg/mL) and incubated at 55°C for 1 h. After addition of 2 mL 3 M sodium acetate, the sample was mixed with 20 mL phenol/chloroform by gently rotating for 30 min. After centrifugation at 4500 rpm for 20 min, the aqueous phase was divided into a few tubes. Each tube was added with 3 volume 100% ethanol and centrifuged at 4500 rpm for 20 min, and the pellet was dried and suspended in 1 mL TE, which was used as the genomic DNA sample.

### 16S ribosomal RNA gene analysis

16S rRNA gene sequence was amplified by PCR from the genomic DNA sample of strain JHA19 using universal primers listed in S1 Table. The DNA sequence of the PCR product was applied to a BLAST search, and the strain species was identified.

### Whole-genome sequencing analysis

Whole-genome shotgun sequencing of the strain JHA19 was conducted using an FLX454 sequencer (Illumina). As a result, 252 Mbp was generated from 6x10^5^ sequencing reads, which gave 32.7 fold-coverage. For sequence assembling, the program Newbler version 2.7 was used, and 70 contigs were generated. The genome annotation was performed with both Glimmer version 3.02b and BLAST 2.2.26. More detailed information will be presented elsewhere.

### Preparation of recombinant Gal*f*-ase proteins

To construct recombinant expression plasmids, four candidate Gal*f*-ase genes were amplified by PCR using the DNA polymerase PrimeStarGXL (Takara), primers shown in S1 Table and genomic DNA of JHA19 as a template. An *Eco*RI digested pET50b vector and amplified DNA were ligated with In-Fusion HD Cloning Kit (Takara).

*Escherichia coli* BL21(DE3)CodonPlus strain transformed with each Gal*f*-ase expression plasmid was precultured in LB medium (Miller, Merck) at 37°C overnight. OD_600_ of cells was adjusted to 0.05 and cultured until OD_600_ = 0.8, added with 100 mM IPTG and cultured overnight at 15°C. Cells were centrifuged at 7000 rpm for 7 min, resuspended in 5 mL 20 mM MOPS (pH 8.0) and lysed by ultrasonication on ice. The cell lysates were centrifuged at 15000 rpm for 10 min at 4°C and the supernatants were applied to a HisTrapTM FF 1 mL column (GE Healthcare). Recombinant protein purification was performed according to the manufacturer’s instructions.

### Preparation of galactomannan from *Aspergillus fumigatus*

Galactomannan (GM) was prepared from *A*. *fumigatus* essentially as described previously with some modifications [27]. Conidia were harvested from a plate of minimal medium (1% glucose, 0.6% NaNO_3_, 0.052% KCl, 0.052% MgSO_4_･7H_2_O, 0.152% KH_2_PO_4_, biotin (trace) and Hunter’s trace elements, pH 6.5), where the *A*. *fumigatus* A1163 (CEA10) strain was grown at 37°C for 3 days. The collected conidia were inoculated in a 500 mL Sakaguchi flask with 100 mL YNB medium supplemented with galactose (YNBG medium; 0.67% yeast nitrogen base, 0.5% (NH_4_)_2_SO_4_, 9% galactose) and precultured at 37°C for 24 h. The preculture was transfered in a 5 L round-bottom flask with 1 L YNBG medium and cultured at 37°C for 14 days. Thereafter, cells from 4.4 L culture were added with formaldehyde at a final concentration of 1% and left for 24 h. After centrifugation, the supernatant was dialyzed with water for 3 days, then evaporated and lyophilized. The resultant sample was dissolved in 5 mL 20 mM phosphate buffer (pH 7.0), applied to TOYOPEARL DEAE-650 (TOSOH) and sequentially eluted with water, 0.5 M and 1 M NaCl solutions in 20 mM phosphate buffer (pH 7.0). The water eluate was dialyzed with 10 mM and 5 mM phosphate buffer (pH 7.0) and water overnight, for 6 h and 1 h, respectively. The resultant solution was evaporated and lyophilized, then used as the GM sample.

### TLC analysis

N-terminal tags (2xHis_6_ and Nus) were cleaved off the recombinant ORF1110 protein using HRV3C protease (Novagen) and removed by chromatography on a HisTrapTM FF 1 mL column. The flow-through sample was concentrated to 22.5 μL (7.6 mU/μL) and incubated with 25 μL GM (1 mg/μL) and 2.5 μL acetate buffer (1 M, pH 4.5) at 37°C for 24 h. The sample was then separated by TLC using a TLC Silica gel 60 plate (Millipore) and 1-butanol/ethanol/water (2:1:1, v/v/v) as solvent. For detection the TLC plate was sprayed with 0.2% orcinol and 10% methanol/sulfuric acid and baked at 120°C for 10 min.

### ELISA

To analyze Gal*f*-ase activity of the ORF1110 protein by ELISA, Platelia *Aspergillus* Ag EIA Kit (Bio-Rad) was used according to the manufacturer’s instructions. Briefly, 50 μL of positive control containing GM, 0.5 μL of 7.5 mU ORF1110 Gal*f*-ase and 1 μL of acetate buffer (1 M, pH 4.5) were mixed in a total volume of 100 μL and incubated at 37°C for 0, 1, 3 or 6 h. The resultant samples were diluted four times and their absorbance at 450 nm was measured.

### Bioinformatic analysis

BLAST (http://blast.ncbi.nlm.nih.gov/Blast.cgi) searches were conducted using sequences of 16S rRNA gene or ORF1110 Gal*f*-ase of strain JHA19. Retrieved sequences were subjected to the program CLUSTAL W (http://clustalw.ddbj.nig.ac.jp/index.php?lang=ja) [42] and their clustering was performed using the neighbor-joining method. Domain search for amino acid sequences was performed using the program Pfam (http://pfam.xfam.org/). Prediction of GH family was carried out using the program CAT (http://mothra.ornl.gov/cgi-bin/cat/cat.cgi?tab=Home).

### Accession numbers

ORF0232, ORF1110, ORF2125 and ORF2812 have been deposited at DDBJ/EMBL/GenBank under the accession nos. LC073693, LC073694, LC073695 and LC073696, respectively.

## Results

### Identification of a soil microorganism that exhibits Gal*f*-ase activity

To search for a Gal*f*-specific Gal*f*-ase, we isolated 282 bacterial strains, mainly actinomycetes, from soil samples. Culture supernatants of three isolated strains, named JHA19, JHA26 and EMA216, exhibited Gal*f*-ase activity using *p*NP-β-D-Gal*f* as a substrate. In addition to the Gal*f*-ase activity, we detected the activities of β-galactosidase (pyranose form), α-mannosidase, β-*N*-acetylgalactosaminidase and β-*N*-acetylglucosaminidase from the culture supernatant of JHA19 using the corresponding *p*NP-glycosides as substrates. Since the activity of Gal*f*-ase was higher than that of Ara*f*-ase, which was hardly detected in the culture supernatant of JHA19, it suggested that this strain might harbor enzyme(s) specific for Gal*f*-ase. Therefore, we chose strain JHA19 for further enzymatic characterization.

**Fig 1:**
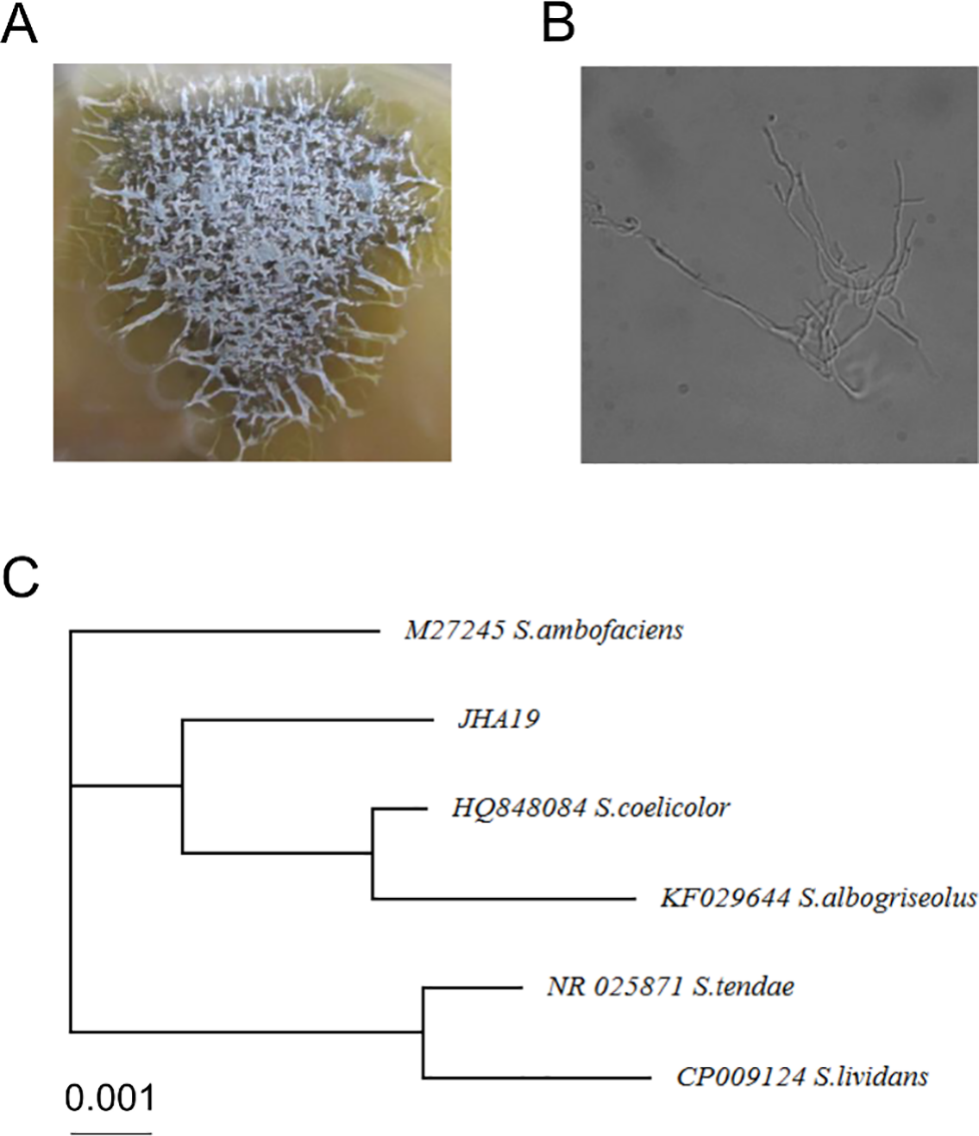
Identification of the strain JHA19. Strain JHA19 (A) grown on a plate and (B) observed under microscope. (C) Phylogenetic tree of 16S rRNA gene sequences from *Streptomyces* species constructed using the neighbor-joining method. The 16S rRNA gene sequence of strain JHA19 was amplified by PCR using universal primers and genomic DNA as a template, determined by DNA sequencing and then subjected to the program CLUSTAL W for the phylogenetic analysis. The DDBJ accession numbers of the sequences used for phylogenetic comparisons are depicted.

Strain JHA19 displayed filamentous growth on a plate (Fig 1A) and appeared like a Gram-positive and bacillary bacterium (Fig 1B), suggesting that it belongs to the *Streptomyces* species. To further identify this strain, we performed a BLAST search based on the 16S rRNA gene sequence, and found that it shows 99% identity to *Streptomyces coelicolor*, *S*. *albogriseolus*, *S*. *tendae*, *S*. *ambofaciens* and *S*. *lividans* (Fig 1C). This result clearly demonstrated that strain JHA19 belongs to the *Streptomyces* species.

### Exploration of candidate Gal*f*-ase genes in strain JHA19

**Fig 2:**
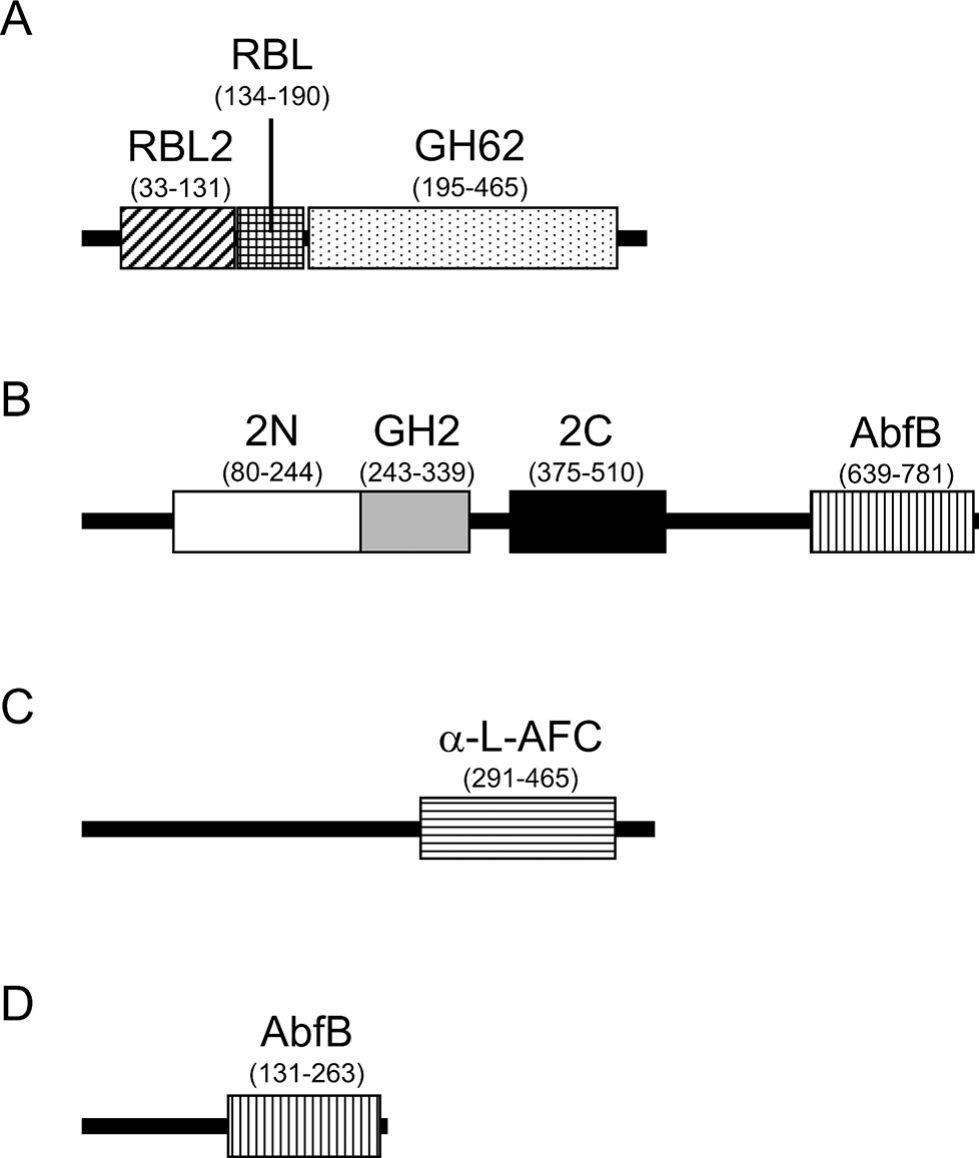
Domains of ORF0232, 1110, 2125 and 2812 proteins. The program Pfam was used to predict domains on each ORF protein. The corresponding amino acid numbers of each domain are indicated in parenthesis. (A) ORF0232: RBL2, Ricin-type beta-trefoil lectin domain-like; RBL, Ricin-type beta-trefoil lectin domain; GH62, Glycosyl hydrolases family 62 domain. (B) ORF1110: 2N, Glycosyl hydrolases family 2, sugar binding domain (Galactose-binding domain-like superfamily); GH2, Glycosyl hydrolases family 2 domain; 2C, Glycosyl hydrolases family 2, TIM barrel domain (Tim barrel glycosyl hydrolase superfamily); AbfB, α-L-arabinofuranosidase B domain. (C) ORF2125: α-L-AFC, α-L-arabinofuranosidase C-terminus domain. (D) ORF2812: AbfB, α-L-arabinofuranosidase B domain.

To search for genes encoding Gal*f*-ases, we conducted a whole-genome shotgun sequencing of strain JHA19. We determined most of the genome sequence, the details of which will be reported elsewhere. We searched the sequence for ORFs that showed high sequence similarity to known furanosidase genes and found four Gal*f*-ase candidates named ORF0232, ORF1110, ORF2125 and ORF2812 (Fig 2; Table 1). Based on a domain search using Pfam, we predicted that ORF0232, ORF2125 and ORF2812 may have Ara*f*-ase activity because they show the highest similarity to reported Ara*f*-ases. Indeed, the ORF0232 protein includes glycosyl hydrolases family 62 domain whose known activity is Ara*f*-ase, the ORF2125 protein contains an Ara*f*-ase C-terminus domain and the ORF2812 protein also has an Ara*f*-ase B domain (AbfB), which is typically seen in GH54 Ara*f*-ases [31,37,43]. Furthermore, a BLAST search revealed that ORF1110 has the highest similarity to a gene encoding an uncharacterized GH2 family protein which contains an AbfB domain based on the program CAT. Therefore, we further analyzed these four candidate genes, including ORF1110.

**Table 1:** Candidate genes for β-D-galactofuranosidase in strain JHA19.

ORF	Homolog^a^	Identity (%)	GH^b^	Size (aa)
0232	α-L-arabinofuranosidase [WP_037890671.1 (*Streptomyces viridochromogenes*)]	86	62	495
1110	hydrolase [WP_030950552.1 (*Streptomyces* sp. NRRL F-5140)]	91	2	786
2125	α-N-arabinofuranosidase [XP_010042338.1 (*Streptomyces chartreusis*)]	86	51	502
2812	α-L-arabinofuranosidase [WP_030948980.1 (*Streptomyces* sp. NRRL F-5140)]	59	-	268

^a^ Based on BLAST searches using the amino acid sequences of the four ORFs, the corresponding homologs with the highest degree of identity are shown.

^b^ Based on CAT program predictions.

### Enzymatic activities of recombinant proteins

**Fig 3:**
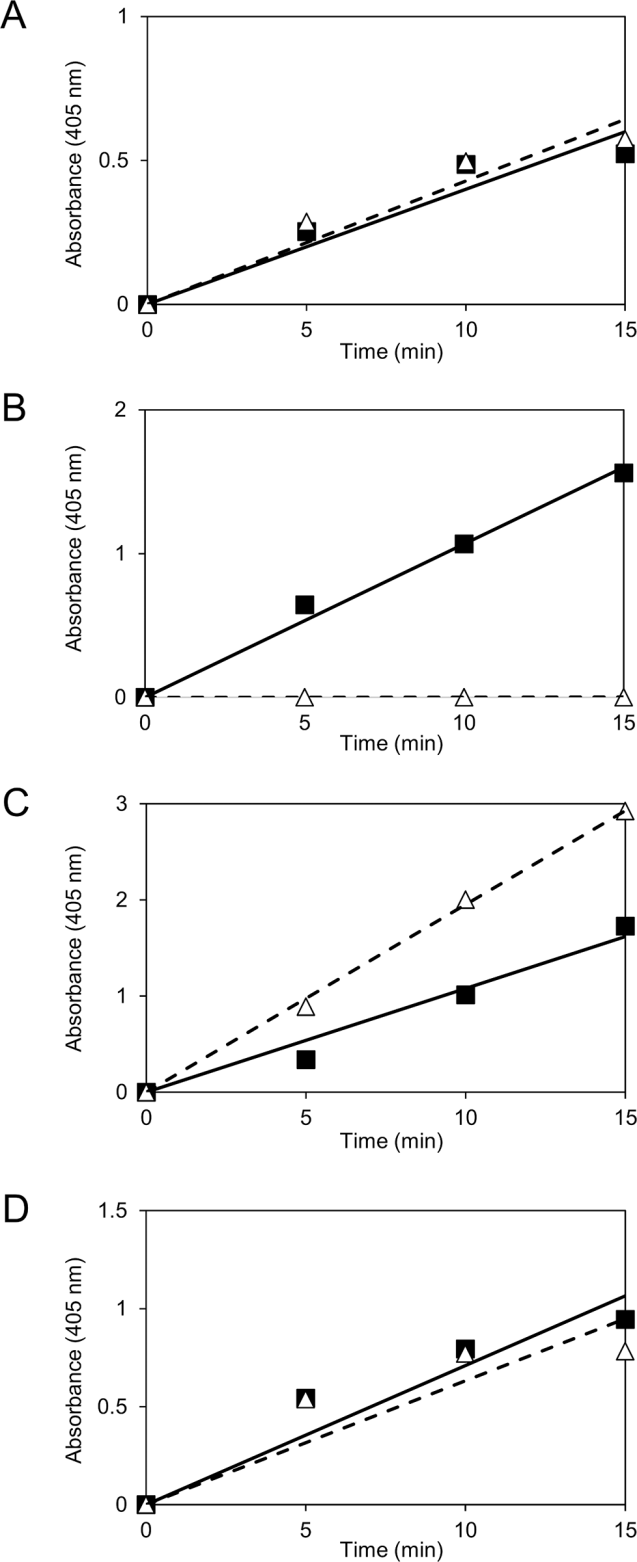
Gal*f*-ase and Ara*f*-ase activities of recombinant proteins. Gal*f*-ase and Ara*f*-ase activities were determined using *p*NP-β-D-Gal*f* (closed square, solid line) and *p*NP-α-L-Ara*f* (open triangle, dotted line) as substrates, respectively. The recombinant proteins of each ORF were used; (A), ORF0232; (B), ORF1110; (C), ORF2125; (D), ORF2812. Note that only the ORF1110 protein showed Gal*f*-ase specific activity. The activity ratios of Ara*f*-ase to Gal*f*-ase are as follows: ORF0232, 1.1:1; ORF2125, 1.8:1; ORF2812, 0.89:1.

We introduced ORF0232, ORF1110, ORF2125 and ORF2812 sequences into an *E*. *coli* expression vector lacking *lacZ* to circumvent a potential risk of contamination of subsequent enzymatic assays by β-galactosidase. The recombinant proteins were expressed and purified by a Ni affinity column. We first confirmed that samples from *E*. *coli* cells harboring an empty vector had no enzymatic activity for *p*NP-α-L-Ara*f* nor *p*NP-β-D-Gal*f* (data not shown). Recombinant proteins expressed from ORF0232, ORF2125 and ORF2812 showed Ara*f*-ase activity for *p*NP-α-L-Ara*f* as a substrate, like their homologs (Fig 3A, 3C and 3D). In addition, we measured the ratio of the activity of Ara*f*-ase to Gal*f*-ase, and found that ORF0232, ORF2125 and ORF2812 proteins exhibited the activity for both Ara*f*-ase and Gal*f*-ase. AbfA and AbfB in *A*. *niger* also showed both Ara*f*-ase and Gal*f*-ase activities, but the activity of Gal*f*-ase was 10-fold less than that of Ara*f*-ase, unlike proteins of ORF0232, ORF2125 and ORF2812 [30]. Although homologs of ORF0232, ORF2125 and ORF2812 are reported as Ara*f*-ases, these recombinant proteins also displayed the Gal*f*-ase activity, suggesting that enzymes reported as Ara*f*-ases might generally exhibit the Gal*f*-ase activity. In contrast, the recombinant protein of ORF1110 exhibited Gal*f*-ase activity only, but not Ara*f*-ase activity, suggesting that this GH2 family protein is a Gal*f*-specific Gal*f*-ase (Fig 3B). Thus, we focused on examining chemoenzymatic characteristics of the ORF1110 protein.

### Chemoenzymatic properties of ORF1110 encoded Gal*f*-ase

To determine the substrate specificity of the recombinant ORF1110 protein, we measured hydrolytic activity using a variety of *p*NP-glycosides in their pyranose form (β-D-Gal, α-D-Gal, β-D-Glc, β-D-Man, α-D-Man, β-D-Xyl, α-D-Xyl, β-D-GalNAc and β-D-GlcNAc). No activity was observed with any of these substrates, except with *p*NP-β-D-Gal*f*, confirming that this enzyme specifically hydrolyzes β-D-Gal*f*.

**Fig 4:**
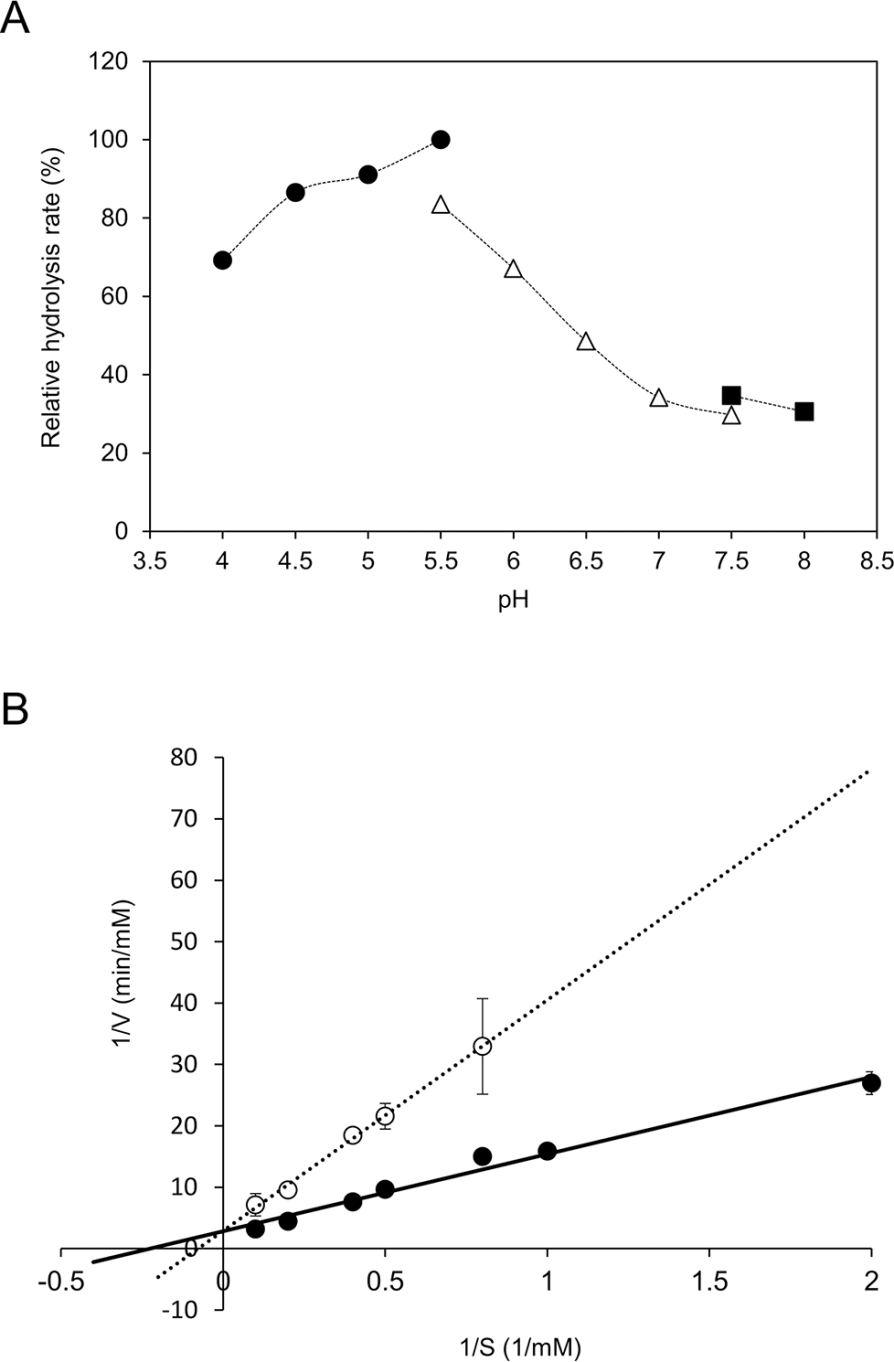
Optimum pH and inhibitory effect of L-arabino-1,4-lactone on Gal*f*-ase activity of ORF1110 protein. (A) The optimum pH of ORF1110 protein for Gal*f*-ase activity was determined using buffers of sodium acetate-acetic acid (4–5.5, closed circle), potassium phosphate (5.5–7.5, open triangle) and MOPS (7.5–8, closed square). (B) Competitive inhibition of Gal*f*-ase activity by L-arabino-1,4-lactone was analyzed with (open circle) or without (closed circle) the inhibitor. Each data point indicates the mean with standard deviation (error bars) from three experiments.

The optimum pH for ORF1110 Gal*f*-ase activity was found to be 5.5 (Fig 4A). The thermal stability of the enzyme was examined by heating it at various temperatures for 10 min. The enzyme was found to be stable at temperatures up to 40°C.

The activity of the Ara*f*-ase TtAFase belonging to the GH2 family in *Thermotoga thermarum* was reported to be highly inhibited by addition of either Cu^2+^ or Zn^2+^ [44]. Hence, we investigated the effects of metal ions (at a concentration of 5 mM) on the Gal*f*-ase activity of ORF1110. We found that the ORF1110 Gal*f*-ase activity was mostly inactivated by addition of Cu^2+^, Zn^2+^ and EDTA to 6.5%, 49% and 55% of its original activity, respectively.

Next, we examined the effect of the substrate *p*NP-β-D-Gal*f* concentration on the initial velocity of the enzyme reaction. The apparent Km and Vmax were 4.4 mM and 0.35 mM/min, respectively. Even though this protein does not have Ara*f*-ase activity, it exhibited competitive inhibition by L-arabino-1,4-lactone, an Ara*f*-ase inhibitor (Ki, 51 mM) (Fig 4B) [45]. This suggests that there may be different substrate recognition mechanism between Gal*f*-ase and Ara*f*-ase at the active site.

### Crucial amino acid residues of ORF1110 Gal*f*-ase

The recombinant ORF1110 protein lacking an AbfB domain exhibited almost the same Gal*f*-ase activity as the full-length protein, suggesting that this domain is likely not required for the Gal*f*-ase activity (data not shown).

**Fig 5:**
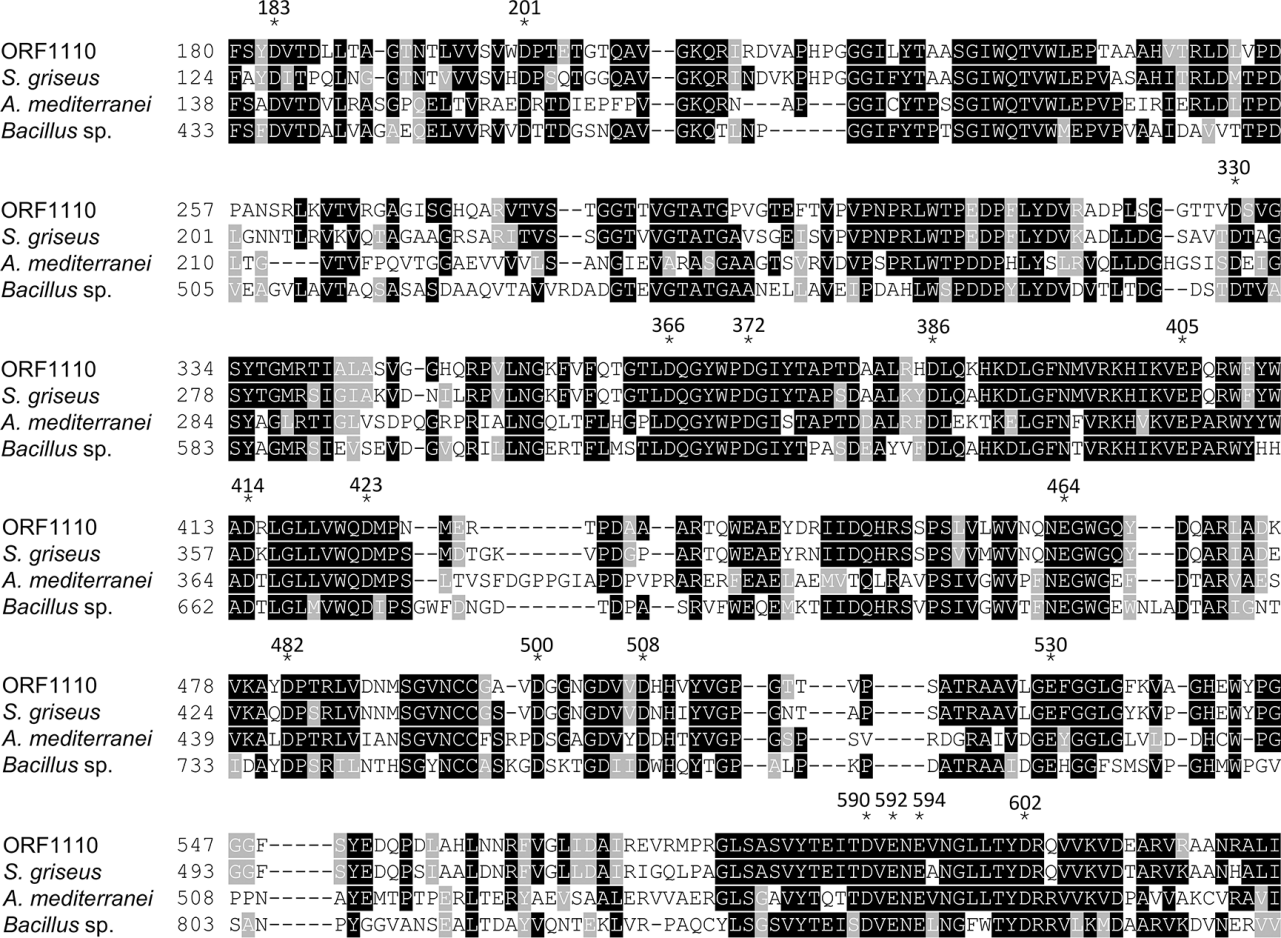
Alignment of ORF1110 Gal*f*-ase and its homologs. Alignment of partial ORF1110 Gal*f*-ase sequence and corresponding regions of its homologs is shown. The conserved amino acid residues indicated with asterisks were mutated into alanine. The DDBJ accession numbers are as follows: *S*. *griseus*, WP012382063; *A*. *mediterranei*, WP013223544; *Bacillus* sp., WP031315417.

Since the protein encoded by ORF1110 shows low similarity to well-known Ara*f*-ases, it is not possible to predict which amino acid residues are crucial for its enzymatic activity by sequence comparison. Thus, we first used a sequence alignment of the GH2 family proteins that show higher sequence similarities to the ORF1110 Gal*f*-ase (Fig 5). The sequence alignment revealed a number of conserved aspartic acid and glutamic acid residues. Using site directed mutagenesis we individually changed each conserved residues to alanine in ORF1110 and measured the effect of the mutations on Gal*f*-ase activity. Most mutations had an effect on Gal*f*-ase activity with D423A and E464A having the most drastic effect, suggesting that the glycosyl hydrolase 2C domain is the catalytic center of this enzyme, and that several amino acid residues are involoved (Table 2).

**Table 2:** Relative Gal*f*-ase activity of recombinant wild-type and mutant ORF1110 proteins.

ORF1110 protein	Relative activity (%)^a^
WT	100
D183A	80.7
D201A	15.5
D330A	6.5
D336A	100
D372A	95.1
D386A	118
E405A	9.7
D414A	66.6
D423A	1.8
E464A	2.6
D482A	75.4
D500A	9.8
D508A	33.5
E530A	15.2
D590A	62.7
E592A	54.7
E594A	11.3
D602A	107

^a^ The relative Gal*f*-ase activity was analyzed using purified recombinant proteins and *p*NP-β-D-Gal*f* as a substrate.

The relative activity of the WT protein was set as 100.

### ORF1110 Gal*f*-ase can hydrolyze *Aspergillus fumigatus* GM

**Fig 6:**
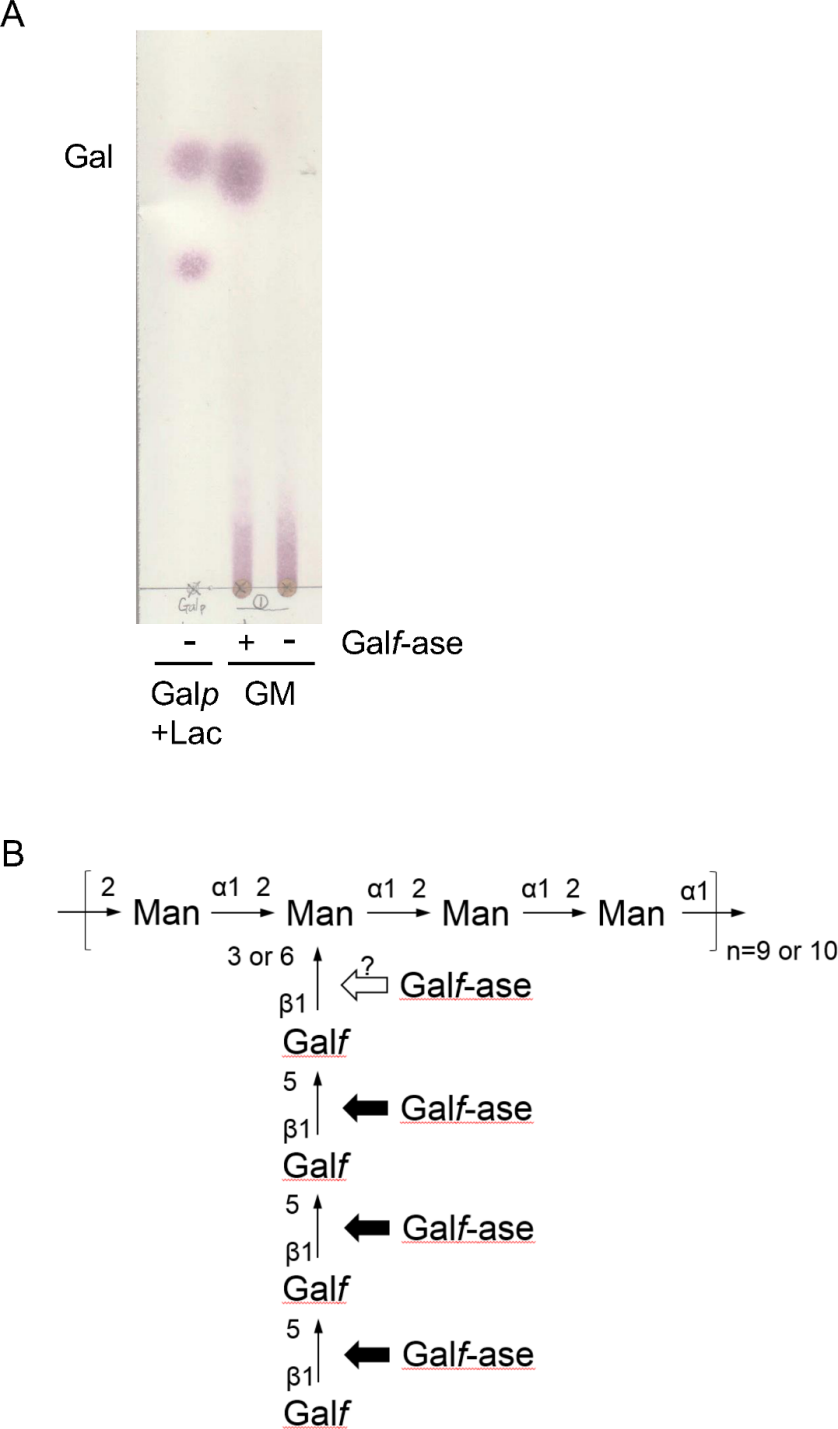
TLC analysis of Gal*f*-ase activity for GM. (A) The recombinant ORF1110 Gal*f*-ase was mixed with GM prepared from *A*. *fumigatus* and incubated at 37°C for 24 h. The resultant sample was subjected to TLC analysis. As references, galactopyranose (Gal*p*) and lactose (Lac) were spotted. As a negative control, a sample without recombinant ORF1110 Gal*f*-ase was spotted. GM, galactomannan. (B) A schematic diagram of the predicted partial structure of *A*. *fumigatus* GM proposed previously [7]. The ORF1110 Gal*f*-ase seems to catalyze terminal Gal*f* residues of *A*. *fumigatus* GM.

Lastly, we tested whether ORF1110 Gal*f*-ase could catalyze not only the artificial substrate *p*NP-β-D-Gal*f* but also a natural Gal*f*-containing oligosaccharide. β-D-Gal*f* exists in glycan parts at the cell surface of *Aspergillus* species. Thus, we extracted GM, including Gal*f* chains, from *A*. *fumigatus* strain A1163 (CEA10) and analyzed Gal*f*-ase activity by TLC (Fig 6A). The results indicated that Gal was released from the GM sample, suggesting that ORF1110 Gal*f*-ase can hydrolyze a natural GM oligosaccharide from *A*. *fumigatus* (Fig 6B).

## Discussion

In this study, we have isolated a strain of *Streptomyces* which possesses Gal*f*-specific Gal*f*-ase encoded by ORF1110. To our best knowledge, this is the first report about Gal*f*-specific Gal*f*-ase that does not also exhibit Ara*f*-ase activity. Since we found that the ORF1110 Gal*f*-ase belongs to GH2, which generally has a β-D-galactosidase activity, we examined hydrolase activity of the ORF1110 enzyme towards *p*NP-β-D-galactopyranoside. However, no activity was detected, suggesting that the ORF1110 enzyme activity is specific to furanose substrates.

**Fig 7:**
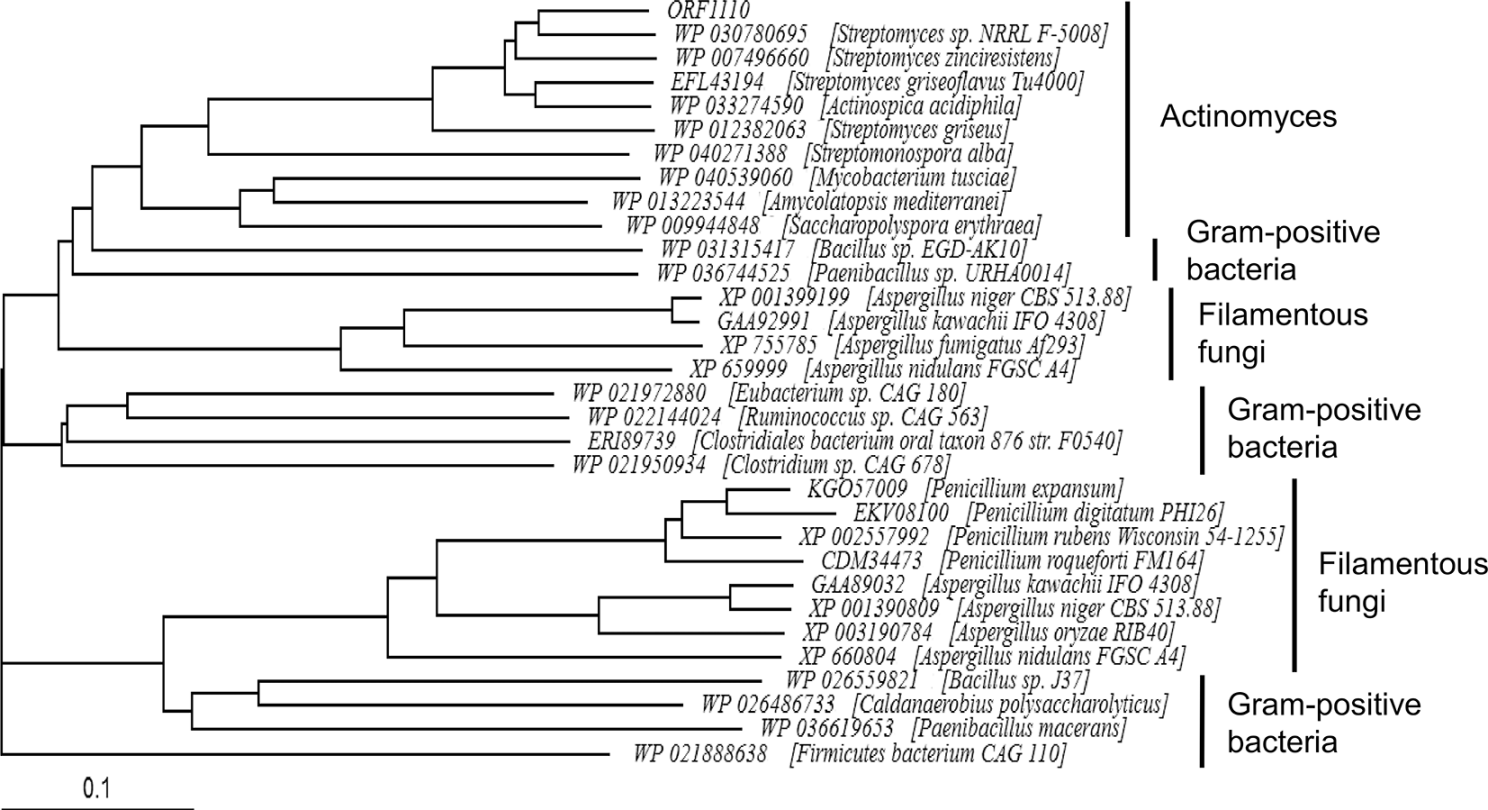
Phylogenetic tree of ORF1110 Gal*f*-ase homologs. The amino acid sequences of hydrolases belonging to the GH2 family were retrieved by a BLAST search using ORF1110 Gal*f*-ase sequence and phylogenetically analyzed using the program CLUSTAL W. The DDBJ accession numbers of sequences used are shown. Note that ORF1110 Gal*f*-ase homologs are widely distributed in Gram-positive bacteria and filamentous fungi.

BLAST search suggested that ORF1110 protein-like Gal*f*-ases exist in a wide range of organisms from bacteria to eukaryotes (Fig 7). We cloned an ORF1110 homologous gene in *Streptomyces griseus* and confirmed that the derived recombinant protein exhibited Gal*f*-specific Gal*f*-ase activity (unpublished data). In *Aspergillus* species, there are also genes corresponding to ORF1110. Since the Gal*f* biosynthetic pathway is important for their hyphal growth, Gal*f*-degradation and metabolism pathways regulated by Gal*f*-specific Gal*f*-ase would be also crucial for fungal physiology. However, little is known about molecular mechanisms of Gal*f*-degradation and metabolism. Therefore, it would be interesting to investigate the physiological functions of genes encoding Gal*f*-specific Gal*f*-ases in *Aspergillus* species.

It was reported that Ara*f*-ases AbfA and AbfB in *A*. *niger*, belonging to the GH51 and GH54, respectively, exhibit activities of both Ara*f*-ase and Gal*f*-ase [30]. Although both *p*NP-β-D-Gal*f* and *p*NP-α-L-Ara*f* are recognized as substrates by AbfB, affinity for *p*NP-β-D-Gal*f* is lower resulting in less Gal*f*-ase activity compared to Ara*f*-ase. Considering that the ORF1110 protein exhibits only Gal*f*-ase activity, almost no Ara*f*-ase activity, and shows the competitive inhibition by L-arabino-1,4-lactone, the C6 atom of Gal*f* in the substrate *p*NP-β-D-Gal*f* appears to be crucial in the hydrogen bonding required for the proper positioning of the substrate on the catalytic site. *p*NP-α-L-Ara*f*, which structure is similar to that of *p*NP-β-D-Gal*f*, would enter the active site of the ORF1110 Gal*f*-ase, but *p*NP-α-L-Ara*f* may exhibit less hydrogen bonding due to lack of the C6 atom, resulting in lower activity of Ara*f*-ase than Gal*f*-ase. The structural analysis of the ORF1110 Gal*f*-ase will be required to reveal the details of the catalytic mechanism.

We confirmed the Gal*f*-ase activity of the ORF1110 protein for *A*. *fumigatus* GM in two ways: One was by detecting non reducing terminal Gal*f* by TLC analysis, and the other was by observing a 40% reduction in ELISA assays using EB-A2 antibody (data not shown). Although we could not find to analyze as a candidate Gal*f*-ase, we found another putative hydrolase gene adjacent to ORF1110 in the JHA19 genome. This predicted hydrolase belongs to GH2 and contains signal peptide like the ORF1110 Gal*f*-ase. These information suggests that this putative hydrolase might be simultaneously expressed with ORF1110 to function together with the ORF1110 Gal*f*-ase. Using *A*. *fumigatus* GM as a substrate, it was shown that the culture supernatant of *A*. *fumigatus*, unlike ORF1110 Gal*f*-ase, produced several bands on TLC, suggesting that there might be not only exo-Gal*f*-ase but also endo-Gal*f*-ase activity in the fungus [27]. In addition, a detailed structural analysis of the sugar chain on glycoproteins demonstrated that β-1,2- and β-1,6-linked Gal*f*, except for β-1,5-linked Gal*f*, also exist [27]. Further work will be needed to determine which linkage of Gal*f* is hydrolyzed by ORF1110 Gal*f*-ase.

In conclusion, we have characterized a novel Gal*f*-specific Gal*f*-ase encoded by ORF1110 in strain JHA19. Considering that ORF1110 Gal*f*-ase homologs are widely present and Gal*f* residues are present on the cell surface of pathogenic microbes such as *A*. *fumigatus*, it is crucial to further understand the molecular mechanisms driving Gal*f*-catalyzing enzymes for establishing novel pharmaceutical therapy against fungal pathogens.

## Supporting Information

S1 Table Primers used in this study. (DOCX)
