# Supplementary material for: Identification and Characterization of a Novel Galactofuranose-Specific β-D-Galactofuranosidase from Streptomyces Species
Source: PLoS One. 2015 Sep 4;10(9):e0137230. doi: 10.1371/journal.pone.0137230 (PMC4560423; doi:10.1371/journal.pone.0137230)
Supplement: S1 Table — (DOCX) [file pone.0137230.s001.docx]

**Supporting Information**

**Supplementary Table 1. Primers used in this study.**

| Target | Sequence (5’ to 3’; upper, forward; lower, reverse) |
| --- | --- |
| 16S rRNA gene | AGAGTTTGATCMTGGCTCAG (M: A or C)  GGYTACCTTGTTACGACTT (Y: A, T or C) |
| ORF0232 | ATGTGGTCACGCATCCTGCCCGCGGTGCGC  TCAGCGCTGCAAGGTCAGCAGGCCCGGGCG |
| ORF1110 | ATGCGTCGAGTACCCGCAGGCCGGCAGAGG  TCATGGCGCCCAGGGCGCCTCCACGGCCCA |
| ORF2125 | ATGCGTACCGCCCGCTTCACCCTCGACCCC  TCAGGCCAGCCGGATCACGTTCCAGGAGAG |
| ORF2812 | ATGCCCGACAACGCATCCAGGTCACCCCAG  TCATGGGGCGGGTGAGTCGACCAGCCGGAA |
| ORF0232  (for In-Fusion) | *accaggatccgaattct*ATGTGGTCACGCATCCTGCC  *ggcctgtacagaatt*TCAGCGCTGCAAGGTCAGCA |
| ORF1110  (for In-Fusion) | *accaggatccgaattct*ATGCGTCGAGTACCCGCAGGCC  *ggcctgtacagaatt*TCATGGCGCCCAGGGCGCCT |
| ORF2125  (for In-Fusion) | *accaggatccgaattct*ATGCGTACCGCCCGCTTCAC  *ggcctgtacagaatt*TCAGGCCAGCCGGATCACGT |
| ORF2812  (for In-Fusion) | *accaggatccgaattct*ATGCCCGACAACGCATCCAG  *ggcctgtacagaatt*TCATGGGGCGGGTGAGTCGA |
| D183A | TCCTACGcCGTCACCGACCTGCTCACC  GGTGACGgCGTAGGAGAAGGCGTCGTA |
| D201A | GTCTGGGcCCCCACCGAGACCGGCACC  GGTGGGGgCCCAGACGGAGACGACCAG |
| D330A | ACCGTCGcCTCCGTCGGCAGCTACACC  GACGGAGgCGACGGTGACGGTGGTGCC |
| D366A | ACGCTCGcCCAGGGCTACTGGCCCGAC  GCCCTGGgCGAGCGTGCCGGTCTGGAA |
| D372A | TGGCCCGcCGGCATCTACACCGCCCCC  GATGCCGgCGGGCCAGTAGCCCTGGTC |
| D386A | CGCCACGcCCTGCAGAAGCACAAGGAC  CTGCAGGgCGTGGCGCAGGGCGGCGTC |
| E405A | AAGGTCGcACCACAGCGCTGGTTCTAC  CTGTGGTgCGACCTTGATGTGCTTGCG |
| D414A | TGGGCCGcCCGGCTGGGACTGCTGGTC  CAGCCGGgCGGCCCAGTAGAACCAGCG |
| D423A | TGGCAGGcCATGCCGAACATGGAGCGC  CGGCATGgCCTGCCAGACCAGCAGTCC |
| E464A | CAGAACGcGGGCTGGGGCCAGTACGAC  CCAGCCCgCGTTCTGGTTGACCCACAG |
| D482A | GCGTACGcCCCCACCCGGCTCGTCGAC  GGTGGGGgCGTACGCCTTGACCTTGTC |
| D500A | GCCGTCGcCGGCGGCAACGGGGACGTC  GCCGCCGgCGACGGCACCGCAGCAGTT |
| D508A | GTCGTCGcCCACCATGTCTACGTCGGC  ATGGTGGgCGACGACGTCCCCGTTGCC |
| E530A | CTCGGCGcGTTCGGCGGCCTGGGCTTC  GCCGAACgCGCCGAGGACGGCGGCGCG |
| D590A | ATCACGGcCGTCGAGAACGAGGTCAAC  CTCGACGgCCGTGATCTCGGTGTAGAC |
| E592A | GACGTCGcGAACGAGGTCAACGGCCTG  CTCGTTCgCGACGTCCGTGATCTCGGT |
| E594A | GAGAACGcGGTCAACGGCCTGCTCACC  GTTGACCgCGTTCTCGACGTCCGTGAT |
| D602A | ACCTACGcCCGTCAGGTGGTCAAGGTC  CTGACGGgCGTAGGTGAGCAGGCCGTT |

Italicized lowercase characters represent sequences required for In-Fusion reaction.

Lowercase characters represent nucleotides where point mutations were introduced.

ORF1110 sequence was used as a template for primers of all point mutants.
